# Similarity Queries for Temporal Toxicogenomic Expression Profiles

**DOI:** 10.1371/journal.pcbi.1000116

**Published:** 2008-07-18

**Authors:** Adam A. Smith, Aaron Vollrath, Christopher A. Bradfield, Mark Craven

**Affiliations:** 1 Department of Computer Science, University of Wisconsin, Madison, Wisconsin, United States of America; 2 Department of Biostatistics and Medical Informatics, University of Wisconsin, Madison, Wisconsin, United States of America; 3 Department of Oncology, University of Wisconsin, Madison, Wisconsin, United States of America; Max-Planck-Institut für Informatik, Germany

## Abstract

We present an approach for answering similarity queries about gene expression time series that is motivated by the task of characterizing the potential toxicity of various chemicals. Our approach involves two key aspects. First, our method employs a novel alignment algorithm based on time warping. Our time warping algorithm has several advantages over previous approaches. It allows the user to impose fairly strong biases on the form that the alignments can take, and it permits a type of local alignment in which the entirety of only one series has to be aligned. Second, our method employs a relaxed spline interpolation to predict expression responses for unmeasured time points, such that the spline does not necessarily exactly fit every observed point. We evaluate our approach using expression time series from the Edge toxicology database. Our experiments show the value of using spline representations for sparse time series. More significantly, they show that our time warping method provides more accurate alignments and classifications than previous standard alignment methods for time series.

## Author Summary

We are developing an approach to characterize chemicals and environmental conditions by comparing their effects on gene expression with those of well characterized treatments. We evaluate our approach in the context of the Edge (Environment, Drugs, and Gene Expression) database, which contains microarray observations collected from mouse liver tissue over the days following exposure to a variety of treatments. Our approach takes as input an unknown query series, consisting of several gene-expression measurements over time. It then picks out treatments from a database of known treatments that exhibit the most similar expression responses. This task is difficult because the data tends to be noisy, sparse in time, and measured at irregular intervals. We start by reconstructing the unobserved parts of the series using splines. We then align the given query to each database series so that the similarities in their expression responses are maximized. Our approach uses dynamic programming to find the best alignment of each pair of series. Unlike other methods, our approach allows alignments in which the end of one of the two series remains unaligned, if it appears that one series shows more of the expression response than the other. We finally return the best match(es) and alignment(s), in the hope that they will help with the query's eventual characterization and addition to the database.

## Introduction

Characterizing and comparing temporal gene expression responses is an important computational task for answering a variety of questions in biological studies. We present an approach for answering similarity queries about gene expression time series that is motivated by the task of characterizing the potential toxicity of various chemicals. Our approach is designed to handle the plethora of problems that arise in comparing gene expression time series, including sparsity, high-dimensionality, noise in the measurements, and the local distortions that can occur in similar time series.

The task that we consider is motivated by the need for faster, more cost-efficient protocols for characterizing the potential toxicity of industrial chemicals. More than 80,000 chemicals are used commercially, and approximately 2,000 new ones are added each year. This number makes it impossible to properly assess the toxicity of each compound in a timely manner using conventional methods. However, the effects of toxic chemicals may often be predicted by how they influence global gene expression over time. By using microarrays, it is possible to measure the expression of thousands of genes simultaneously. It is likely that transcriptional profiles will soon become a standard component of toxicology assessment and government regulation of drugs and other chemicals.

**Figure 1:**
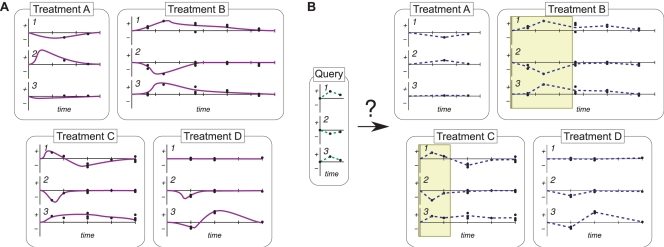
An example of the similarity-query task for four different treatments with three genes. (A) The curves show the actual hidden expression profile for each treatment, even though we must rely on the noisy sampled observations (the dots). (B) We have reconstructed the profiles at unobserved times, and used them to perform a similarity query. The highlighted areas represent possible good matches.

One resource for toxicology-related gene expression information is the Edge (Environment, Drugs, and Gene Expression) database [1]. Edge contains expression profiles from mouse liver tissue following exposure to a variety of chemicals and physiological changes, which we refer to as *treatments*. Some of the treatments in Edge have been assayed as time series. Figure 1A provides a simplified illustration of the type of data with which we are concerned. The small database in this figure contains time series data for four different treatments, each of which includes measurements for three genes. The true, underlying expression response is not known, but instead the database contains sampled observations which may be noisy. We use the term *observation* to refer to the expression measurements made at a single time point in a treatment.

The computational task that we consider is illustrated in Figure 1B. Given an expression profile as a query, we want to identify the treatment in the database that has the expression profile most similar to the query. In the general case, the query and/or some of the database treatments are time series. In this case, we want to also determine the temporal correspondence between queries and putatively similar treatments in the database. In the toxicology domain, we are interested in answering this type of query in order to characterize poorly understood chemicals.

There are several properties of the expression time series at hand that are important considerations for our work.

- *Sparsity:* As is the case with most time series characterizing gene expression [2], the time series available from toxicological studies typically contain measurements from only a handful of time points. The longest time series in the Edge database has observations at only 9 times, and several of the series include only two points.
- *High-dimensionality:* Because the expression data we consider is measured via microarrays, each time “point” in our series lies in a high-dimensional space. For the experiments reported here, each time point represents expression levels for 1,600 genes. (Technically, the expression measurements correspond to clones selected from liver-derived EST and full-length cDNAs. These clones represent products for 1,600 unique genes.)
- *Non-uniform and irregular sampling:* Given the sparsity of the time series, it is typically the case that they have been sampled at non-uniform time intervals. Moreover, the sampling times may vary for different time series.
- *Noise:* As is the case with all microarray data, the measurements involve a fair amount of noise due to technical issues in the process.
- *Biological variability:* Because a mouse model is used for the toxicology experiments we consider, there is also a component of biological variation that affects the data measured. Each microarray assays a sample from a different animal.

These properties of the data result in several additional challenges for the task we consider.

- The time points present in a given query may not correspond to measured points in some or any of the time series in the database.
- Queries may be of variable size. Some queries may consist of only a single observation, whereas others may contain multiple time points. Additionally, queries may vary in their extent: some may span only a few hours whereas others include measurements taken over days.
- A given query and its best match in the database may differ in the amplitude, temporal offset, or temporal extent of their responses. For example, the expression profile represented by a query treatment may be similar to a database treatment except that the gene expression responses are attenuated, or occur later, or take place more slowly.
- A given query and its best match in the database may differ in that one of them shows more of the temporal evolution of the treatment responses. In other words, the query may be similar to a truncated version of the database series, or vice versa.

To address these challenges, we have developed a generative model that approaches the problem from a probabilistic perspective. In order to temporally align gene-expression time series using our model, we employ a novel method for *dynamic time warping*. Dynamic time warping [3],[4] is an approach for aligning pairs of time series that was originally developed for speech recognition problems. It employs dynamic programming to find an optimal alignment with respect to a given scoring function. We also use spline interpolation as a preprocessing step to predict expression responses for unmeasured time points, in order to reconstruct a more complete time series.

Our time warping approach differs in several substantial ways from the standard dynamic programming method. Unlike the standard approach, our method does not force the two series to be globally aligned. Instead, it permits a type of *local* alignment in which the end of one series is unaligned. We refer to this case as *shorting* the alignment. This aspect of the approach is motivated by the consideration that one of the series may show more of the temporal response than the other. For example, one series may not have been measured for as long as the other. Another significant way in which our approach differs from standard time warping is that it is based on an explicit, generative model. This model allows the user to explicitly encode costs/probabilities that characterize the likelihood of various types of differences in closely related time series. The most significant way in which our approach differs from standard time warping is that it enables the user to impose fairly strong biases on the form that the alignments can take. In particular, it allows alignments that partition the given time series into a small number of segments in which the changes from one time series to the other (e.g., in terms of amplitude) are fairly uniform. This is important given the sparsity, high-dimensionality, and noisiness of the time series being aligned.

We also investigate variations on spline interpolation in order to find an approach that results in accurate reconstructions of sparsely sampled time series. We find that we achieve more accurate interpolations when using higher order splines. Further, our experiments indicate that it is helpful to relax the splines' fit to the observed data, rather than potentially overfitting by exactly intercepting each observed data point.

In earlier work, our group [5] and others [6] have developed systems for classifying chemicals according to the expression profiles they induce. The approach that we present here differs in that it takes into account the temporal aspects of expression profiles, and it is able to answer similarity queries. The latter property is important because some classes may be very sparsely populated in the database, and class labels may not be available or readily defined for some treatments.

Lamb et al. [7] consider the task of finding expression profiles that are similar to a given query profile, such as one induced by a particular drug. Their approach does not represent time series, however. Moreover, it assumes that the query includes a specified set of genes which are known to be correlated with some state of interest, such as the expression activity induced by the drug. Our approach does not require that such a gene set be provided.

Aach and Church [8] were the first to apply the method of dynamic time warping [3] to gene expression profiles, and other groups have followed [9],[10]. The method we present differs in several key respects. First, our method is able to not only align a pair of time series, but it is also able to pick out the known time series most similar to an unknown one for purposes of classification. Second, we use nonlinear spline models in conjunction with time warping in order to interpolate to unseen time points. Third, we consider local alignments of time series in which one of the series is shorted.

Bar-Joseph et al. [11] have investigated splines and warping in the context of clustering and aligning time series. Our work differs primarily in the task being considered and the use of a more expressive warping model. They restrict their attention to linear warping, whereas we use a “multisegment” model that warps different regions of the series by different amounts.

Listgarten et al. [12] have developed a method for multiple alignment of time series data that has some similarities to our approach. The task they consider—multiple alignment—is different than ours, and their method does not employ splines.

A related approach to aligning time series is proposed by Gaffney and Smyth [13]. They use an expectation-maximization method in concert with a mixture model in order to simultaneously align and cluster time series. Our work, however, is not concerned with clustering known time series. Rather our aim is to use a database of previously seen time series to answer similarity queries about a new one. Further the biases they allow are not appropriate to our task. They allow only linear scaling in time and measurement space whereas we need more complicated warpings, and they allow translation in both these dimensions as well which is unnecessary for us.

Another similar approach is *correlation optimized warping* (COW), devised by Nielsen et al. [14]. They compare time series by dividing them into several roughly equal segments and summing the Pearson's correlations of corresponding segments. The segments may vary in length by up to a slack factor provided by the user, and dynamic programming is used to find the segments with the maximum sum of correlations. Unlike our approach, their method assumes that the series will be globally aligned, without any shorting. Further, the use of correlation can be limiting as COW is unable to distinguish between two series that are proportional to one another.

Our approach is also related to various probabilistic sequence models, such as *generalized* hidden Markov models, that directly evaluate the likelihood of *segments* of a sequence, instead of incrementally computing these likelihoods one sequence element at a time. Models of this type have been used for tasks such as gene finding [15] and secondary structure prediction [16].

## Methods

In this section we detail our generative model for classifying and aligning time series, and present a dynamic programming algorithm that is able to find optimal alignments under this model. We also present a review of B-spline interpolation and discuss some useful variations of the method. We use spline interpolation to reconstruct unobserved microarray observations.

Our approach to answering similarity queries involves three basic steps: (i) we use interpolation methods as a preprocessing step to reconstruct unobserved expression values from our sparse time series; (ii) we use our alignment method to find the highest scoring alignment of the query series to each treatment series in the database; (iii) we return the treatment from the database that is most similar to the query, and the calculated alignment between the two series.

We have implemented all our algorithms in Java. The source code is available for download at http://www.biostat.wisc.edu/aasmith/catcode/.

### Interpolating Expression Profiles with B-Splines

One challenge that arises when aligning a pair of expression time series is that the series may have been sampled at different time points. Moreover, the sampling may be sparse and occur at irregular intervals. To address these issues, we first use an interpolation method to reconstruct the unobserved parts of the time series before trying to align them. This interpolation step allows us to represent each time series by regularly spaced observations. We refer to the “observations” which come from the interpolation, as opposed to measurement, as *pseudo-observations*.

Although linear interpolation is a natural first approximation, other work has explored the use of B-splines to better reconstruct missing expression data [11]. A B-spline is a piecewise polynomial function that is a generalization of a Bézier curve. We present a brief review here, although for depth we refer the reader elsewhere [17].

**Figure 2:**
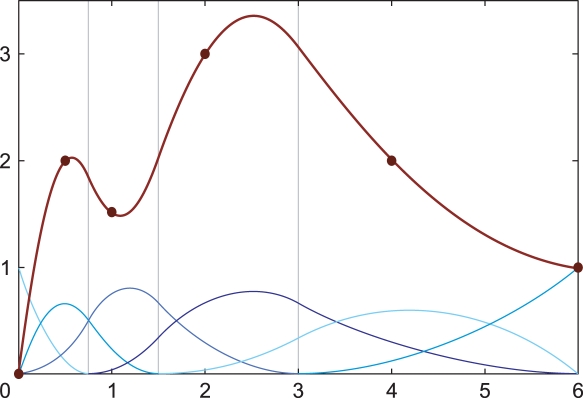
A quadratic B-spline (*k* = 3). The main spline which fits the observed points is a weighted sum of the basis splines shown at the bottom of the figure. These are defined by the Cox-de Boor regression formulas (Equations 2 and 3) in conjunction with pre-defined points of discontinuity (the vertical lines). The weights, called control points, are easily obtained by solving a set of linear equations.

As shown in Figure 2, a B-spline is the weighted sum of a set of basis splines. The basis splines are determined by the desired order *k* of the splines, and the points of discontinuity 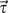 which are called *knots*. There are *n* bases, where:
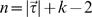
(1)and they are defined via the Cox-de Boor regression formulas:
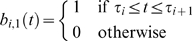
(2)

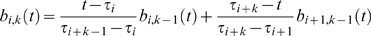
(3)where *b_i_*_,*k*_ is the *i*th base of order *k*.

It follows that the segments of the *k*th-order basis splines have degree of *k*−1, so a second-order B-spline consists of line segments, a third-order spline consists of quadratic segments, etc. The splines are also continuous down to the (k−2)th derivative. The actual interpolating B-spline *s* inherits these properties. It is formally defined as:
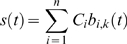
(4)


The weights *C_i_* are known as control points, and solving for them is a simple matter of solving linear equations. With *n* points (*t_i_*,*x_i_*) to interpolate:
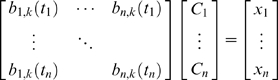
(5)


With fewer than *n* points, the problem is underconstrained and cannot be solved with such a large *k*. With more than *n* points, the problem is overconstrained and can only be solved in a least-squares sense. This is easy to do with standard linear algebra techniques. However, one must make sure that every base overlaps with at least one observation, or the matrix will be rank-deficient and the equations unsolvable.

Unfortunately, B-splines have a tendency to overfit curves in data-impoverished conditions. Such reconstructions can show large oscillations in an attempt to exactly intercept every observed data point. This can be especially problematic with microarray data, which are already inherently noisy. The solution we use is to solve for the control points of a low-order spline, and then use those control points for a higher-order one. Such a spline will tend to fall within the convex hull created by the lower-order spline [17]. We refer to such splines as *smoothing* splines, and refer to B-splines solved with conventional methods as *intercepting* splines.

### A Generative Model for Time Series Alignment

Each possible alignment we consider for two given time series (the query and the database series) partitions the series into *m segments*, where the *i*th segments of the series correspond to one another. Our dynamic programming method tries to find a partitioning of the series that reveals the maximal similarity between them. As discussed earlier, we want to take into account that the nature of the relationship between the two series may vary in different segments. For example, it may be the case that the first part of the expression response occurs more slowly in one treatment than in a similar treatment. Recall also that the segments do not have to cover the entirety of both series—one of the series may be “shorted.”

**Figure 3:**
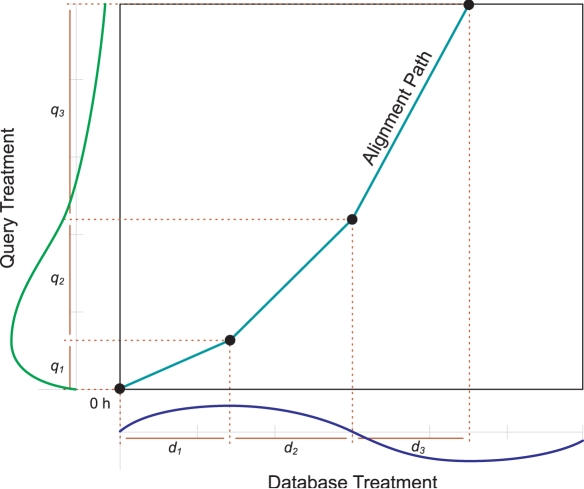
An example of an alignment with local effects. The best alignment between the query treatment and the database treatment being considered involves three segments. The first two segments of the database treatment have increased amplitude, the first segment is contracted (or stretched in), and the third segment is stretched out in order to approximate the observed query treatment. Also the alignment shorts before the database treatment has ended, as there is no evidence that the query treatment expression has begun to increase again at the end.

Figure 3 illustrates the type of alignment we want to consider. This figure shows the optimal alignment between a query treatment and a given treatment in the database. (For simplicity, the figure shows each treatment as consisting of only a single gene.) This alignment involves three different segments, and in each segment the amplitude and stretching relationships between the two series are somewhat different. We use the term *stretching* to refer to distortions in the rate of some response, and the term *amplitude* to refer to distortions in the magnitude of the response. In addition, the alignment has shorted so that the full query is aligned with only a partial database series.

To determine the similarity between a query time series *q* and a particular database series *d*, we can calculate how likely it is that *q* is a somewhat distorted exemplar of the same process that resulted in *d*. In particular, we can think of a generative process that uses *d* to generate similar expression profiles. We can then ask how probable *q* looks under this generative process.

Given this generative process idea, we calculate the probability of a particular alignment of query *q* given a database series *d* as follows:
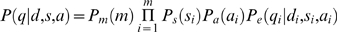
(6)where *m* is the number of segments in the alignment, *q_i_* and *d_i_* refer to the expression measurements for the *i*th query and database segments respectively, and *s_i_* is the stretching value and *a_i_* is the amplitude value for the *i*th segment. The location of each segment pair is assumed to be given here.

*P_m_* represents a probability distribution over the number of segments in an alignment, up to some maximum number *M* of allowed segments. *P_s_* represents a probability distribution over possible stretching values for a pair of segments, *P_a_* represents a probability distribution over possible amplitude values, and *P_e_* represents a probability distribution over expression observations in the query series, given the database series and the stretching and amplitude parameters.

To represent *P_s_*, we use a discretized version of the following distribution:
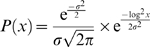
(7)


We choose this distributional form because it is a variation of the log normal distribution that is symmetric around one, such that *P*(*x*) = *P*(1/*x*). Thus for example, stretching some expression response by a factor of two is equiprobable to compressing it by a factor of two. This symmetry property means that it does not matter which series we consider to be the query and which we consider to be from the database. As we discuss in the next section, our dynamic programming algorithm only allows segments to begin and end at a limited number of points. Thus, our distribution is actually discretized so that probability mass is allocated only to possible stretching values, and then renormalized.

We use a similar distribution to represent *P_a_*, the distribution of amplitude values, since we also want to have *P*(*x*) = *P*(1/*x*) symmetry with these values. Thus a twofold increase in an expression response is treated as equiprobable to a twofold decrease.

To calculate *P_e_*(*q_i_*|*d_i_*,*s_i_*,*a_i_*), we transform our representation of *d_i_* using the given stretching and amplitude values, and then ask how probable *q_i_* appears when we use this transformed *d_i_* series as a model. Let us first consider a simple case in which our time series have only one gene, and we are mapping only one point from the query segment *q_i_* to the database segment *d_i_*. Let *t* represent a time coordinate in the segment *q_i_*, and let *q_i_l* and *q_i_r* denote the leftmost and rightmost time coordinates in the *i*th query segment. Let *d_i_l* and *d_i_r* denote the corresponding bounding time coordinates for the *i*th database segment. Then we can map a time coordinate from segment *q_i_* into the corresponding coordinate in *d_i_* as follows:
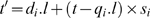
(8)where the stretching value *s_i_* is defined by:
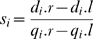
(9)


Our model for “generating” points in the query series from a point in the database series is a Gaussian centered at the database point. Let *p*(*x*,*μ*,*σ_e_*) represent the probability density function of this Gaussian, where *μ* is the mean and *σ_e_* is the standard deviation of the Gaussian. We can then compute the probability of generating a query point *q_i_*(*t*) located at time *t* as:
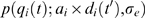
(10)


In other words, we center a Gaussian on the expression level at the mapped time coordinate in the database series, and ask how probable the scaled expression value from the query looks at that time coordinate.

To generalize this calculation to multiple observations in the query series, we make the simplifying assumption that the observations are independent, and we have:
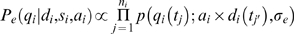
(11)where *n_i_* is the number of query observations in segment *i*.

Each of our observations represents measurements for hundreds of genes. We therefore generalize the description above by having *p*(*x*,*μ*,*σ_e_*) be a multidimensional Gaussian, with one dimension for each gene measured. In our current work, we treat the genes as independent of one another given the time point. Thus the covariance matrix for this Gaussian is zero on all of the off-diagonal terms.

We assume that *σ_e_* represents variation in expression measurements that are due to technical and biological variability. Thus, we estimate the standard deviation for each gene by considering the variance in a sample that consists of all the replicated experiments in the database.

In addition to considering the likelihood of the query series under the assumption that it exhibits a similar response to the given database series, we also consider its likelihood under a null model. The notion of a null model here is one that generates alignments by randomly picking observations from the database to align with the query sequence. The rationale for using such a null model is analogous to the use of a model of *unrelated* sequences in the derivation of substitution matrices for protein sequence alignment [18],[19]. In the case of protein sequence alignment, we want to know the relative likelihood of two cases: one case in which the correspondence between the sequences is explained by their relatedness through evolution, and the alternative in which the sequences are unrelated. In our task, we similarly want to compare the probability of an alignment given a model of relatedness (described above), and an alternative that asks how probable the query would look if we aligned it to an unrelated series.

The value of a null model for our application is that it enables alignments of differing lengths, including shorted alignments, to be compared on an equal footing. Under our scoring function which incorporates the null model, segments have a positive score only if the database series in that segment explains the corresponding segment from the query series better than the null model does.

Let *p*(*x*,*μ_DB_*,*σ_e_*) represent the probability density function of a multidimensional Gaussian whose mean *μ_DB_* is the average expression level of the observations in the database, and whose standard deviation is *σ_e_* as before.

We then estimate the probability of the *i*th segment of the query series under the null model as:
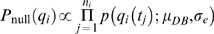
(12)


Since our null model assumes that there is only a single segment with no amplitude change or stretching, we can compute the probability of the entire query series *q* as follows:
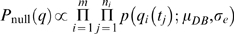
(13)


Putting together the terms above, we can score a given alignment based on the log of the likelihood ratio of the query series under the “database series” model versus the query series under the null model as:
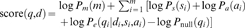
(14)


Up to now we have described this process in terms of using a database series to generate the query series. However, we want our alignment method to be symmetric so that it does not matter which series we consider to be the query and which we consider to be from the database. Due to the last two terms, this will not necessarily be the case using the scoring function defined above. Therefore, we modify the scoring function so that it also considers using the query series to generate the database series:
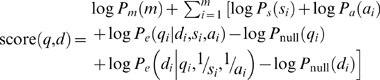
(15)


Here *P_e_*(*d_i_*|*q_i_*,1/*s_i_*,1/*a_i_*) is calculated in an analogous manner to *P_e_*(*q_i_*|*d_i_*,*s_i_*,*a_i_*) but the inverses of *s_i_* and *a_i_* are used to generate observations in the database series.

### A Dynamic Program for Alignment

Given a pair of time series, we do not know a priori which alignment (i.e., placement of corresponding segments) is optimal. However we can find the optimal alignment using dynamic programming. The following algorithm takes as input two time series, termed *q* and *d*, both of which are represented by regularly spaced observations (or interpolated pseudo-observations) of the gene expression values.

In particular, given a segment pair (*q_i_*,*d_i_*), we can calculate its score as follows:
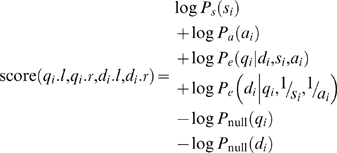
(16)


The arguments to this scoring function define the leftmost and rightmost time coordinates of the segments being aligned from the query series and the database series. These points are selected from the set of regularly spaced observations mentioned above. The stretching parameter, *s_i_* is defined by the relative lengths of the two segments. We find the amplitude coefficient *a_i_* via a least-squares method. Although this least-squares method is not guaranteed to find the optimal value of *a_i_*, we have found that, in practice, it provides solutions comparable to a dense grid search of the parameter, and it is much faster than the latter.

The core of the dynamic program involves filling in a three-dimensional matrix Г in which each element *γ*(*i*,*x*,*y*) represents the best score found with *i* segments that align the query subseries from time 0 to *x* with the database subseries from time 0 to *y*. As above, *x* and *y* must be selected from the given observations in the two series. The basic idea is that in order to determine *γ*(*i*,*x*,*y*), we look through all *γ*(*i*−1,*a*,*b*) where *a*<*x* and *b*<*y*. We then add the score of the segment from (*a*,*b*) to (*x*,*y*) to the value *γ*(*i*−1,*a*,*b*), assigning the best such sum to *γ*(*i*,*x*,*y*).

We define *γ*(*i*,*x*,*y*) with the following recurrence relation:
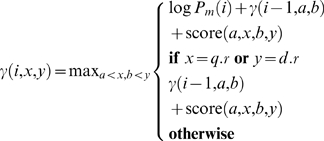
(17)where the base case is:
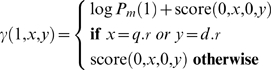
(18)


Here, *q*.*r* and *d*.*r* refer to the rightmost (last) time coordinates in the query series and the database series, respectively. The first condition in each recurrence relation ensures that the distribution over the number of segments *P_m_* is taken into account when we consider the last pair of segments in a candidate alignment.

Recall that we are interested in possibly shorting the alignment, thus finding a local alignment rather than a global one. Allowed alignments are those that explain the entire extent of at least one of the two given time series. In order to recover the optimal alignment, we use a traceback procedure that involves scanning the elements of Г that represent alignments that include the entirety of the query series, the entirety of the database series, or both. The procedure returns the alignment corresponding to the highest-scoring entry among these. More formally, we find the score of the best alignment as follows, and start the traceback from the identified element:
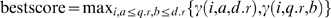
(19)


This dynamic program can be thought of as having three key “penalty terms” that determine the relative scores of alignments. These penalty terms correspond to the probability distributions that govern (i) the number of segments, (ii) the stretching values, and (iii) the amplitude values used in an alignment.

Preferences for the number of segments to be used in alignments are expressed by providing a distribution for *P_m_*. In our work to date, we have assumed a uniform distribution up to the allowed number of segment pairs. It might be valuable to use a distribution that favors fewer segment pairs, however. Preferences for stretching and amplitude values are controlled via the standard deviation *σ* parameter in the distributions over these values. For example, as *σ_a_* for the amplitude distribution is made smaller, a difference in amplitude between the series is penalized more in the scoring scheme.

## Results

In this section we present experiments that evaluate the utility of our novel time warping method and spline models for the task of answering similarity queries with expression profiles.

### Data

The data we use in our experiments comes from the Edge toxicology database [1], and can be downloaded from http://edge.oncology.wisc.edu/. Our data set consists of 216 unique observations of microarray data, each of which represents the expression values for 1,600 different genes. Each of these expression values is calculated by taking the average expression level from four treated animals, divided by the average level measured in four control animals. The data are then converted to a logarithmic scale, so that an expression of 0.0 corresponds to the average basal level observed in the control animals.

Each observation is associated with a treatment and a time point. The treatment refers to the chemical to which the animals were exposed and its dosage. The time point indicates the number of hours elapsed since exposure occurred. Times range from 6 hours up to 96 hours. The data used in our computational experiments span 11 different treatments, and for each treatment there are observations taken from at least three different time points.

We can assume that for all treatments there exists an implicit observation at time zero. This is the time at which the treatment was applied, so all expression values are assumed to be at base level. Therefore every query automatically includes at least two observations: the actual query time(s) and the zero point. Thus earlier points in time can be interpolated, even when there seems to be only a single query observation.

**Figure 4:**
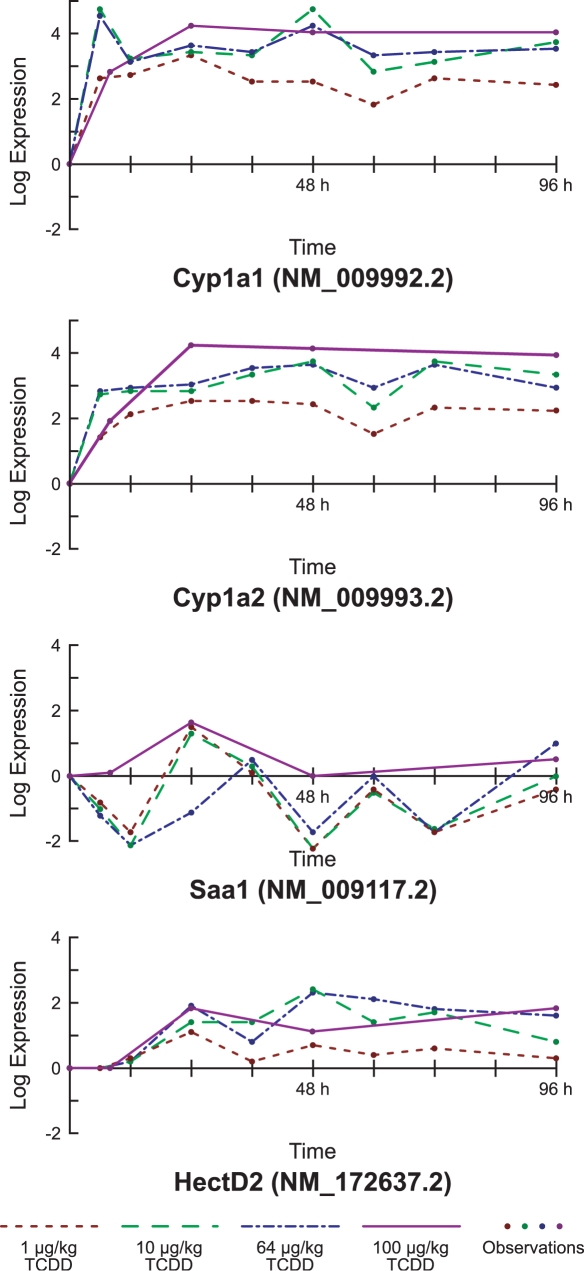
Expression levels of four of the genes most active for TCDD. Linear interpolation is used between these observations of 2,3,7,8-tetrachlorodibenzo-*p*-dioxin, which are represented as points.

Figure 4 illustrates the evolution of four genes over time for 2,3,7,8-tetrachlorodibenzo-*p*-dioxin (TCDD), for four different dosages.

### Interpolating Missing Times

Before we evaluate our generative alignment method, we wish to determine which type of spline (including simple linear interpolation) is the best to use in our preprocessing step. We do this by running a leave-one-out experiment in which we classify each observation in our data set in turn, using the remaining observations as the database. However, we exclude from the database any observation with the same treatment (i.e., chemical and dosage) and time as the query observation. We exclude from the queries observations from the last observed time of each treatment because we cannot interpolate pseudo-observations at these times when they are removed from the database series. We reconstruct hourly pseudo-observations for every treatment, using the different methods of interpolation. We search the reconstructed database for the pseudo-observation that is most like the query. We predict the query's treatment and time to be the same as this nearest neighbor. Notice that by excluding replicates of the query from the database, we are forcing our classifier to use interpolation in order to find the correct answer. We wish to know how accurately we are able to (i) identify the treatment from which each point was extracted, and (ii) align each query point to its actual time in the time series for the treatment. We refer to the former as *treatment accuracy* and the latter as *alignment accuracy*.

We note that this task is only a surrogate for the actual task with which we are concerned—classifying uncharacterized chemicals and aligning them with the most similar treatment in the database. It is a useful surrogate, however, because it is a task in which we know the most similar treatment and the correct alignment of the query to this treatment.

The metric we use to measure distance between the query observation and the database pseudo-observation being considered is a scale-independent Euclidean distance. The expression values of each database observation are all multiplied by a scalar, which is chosen via a least-squares method in order to minimize its distance to the query observation.

We consider seven different interpolation methods in all. We look at both *intercepting* and *smoothing* splines as explained in the Methods section, with orders three, four, and five. The control points for the smoothing splines are based on those for second-order interpolation. We also perform linear interpolation as a control. We use the observed times themselves as our knots (points of discontinuity). If there are too few observation times for a particular order, we use the highest possible order. (For example, if there is only a single observation, we interpolate linearly between it and the implicit zero point, regardless of the overall order used.) To allow for smoothing splines, we must keep the number of bases *n* constant. By Equation 1, the number of knots 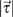 must decrease when the order *k* increases. We do this by resampling them down to the proper number.

There are several advantages to using the observed times as the knots for our interpolating splines. First, it allows easy comparison to the basic linear interpolation control. Second, we assume that the data was taken at those times because interesting behavior was anticipated. Using them as knots allows our splines more flexibility there. Third, it keeps the linear equations from being rank-deficient as explained earlier. With uniformly spaced knots (as used by Bar-Joseph et al. [11]) it is possible to be unable to solve for some control points.

**Figure 5:**
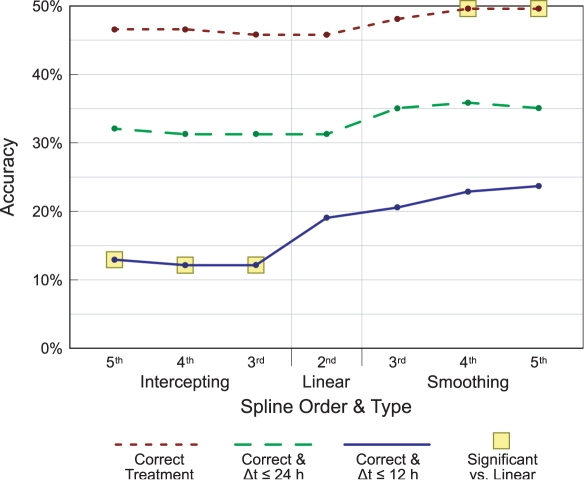
Classification and alignment accuracies resulting from using different B-splines for interpolation. All replicates of the observation tested are purged from the database. The top line shows classification accuracy, in which the correct treatment is chosen. The bottom lines show alignment accuracy, where the predicted time is within 24 and 12 hours respectively of the actual time. Highlighted points are significantly different from the linear case (*p*≤0.05 via McNemar's *χ* ^2^ test).

The results of this experiment are shown in Figure 5. The top line shows classification accuracy, while the lower lines show alignment accuracy—where a case is considered “correct” if in addition to the proper treatment, the predicted time is correct to within 24 or 12 hours respectively. We test the significance of the differences in accuracy (from the linear interpolation control) using McNemar's *χ* ^2^ test. Highlighted points are those deemed significant, with *p*<0.05. For all three accuracy measures we see improvement when using smoothing splines, while intercepting splines perform similarly or worse than the linear interpolation control. The fifth-order smoothing spline has a significantly higher classification accuracy (*p*≈0.025), and also appears to have better alignment accuracy (*p*≈0.132 for Δ*t*≤24 and *p*≈0.180 for Δ*t*≤12). By contrast the more traditional intercepting spline is likely overfitting its interpolation to the limited number of observed times. Although the fifth-order intercepting spline is not significantly different from the linear one for classification accuracy (*p*≈0.739) and alignment accuracy to within 24 hours (*p*≈0.705), there is a noticeable hit in the stricter alignment accuracy (*p*≈0.021). The *p*-values for the lower-ordered splines are qualitatively similar.

Based on these results, we restrict our attention to smoothing splines in subsequent experiments.

### Aligning Time Series

We now turn our attention to evaluating our multisegment time series alignment algorithm. For all of the experiments reported in this section, we set the parameters of this method as follows. We set the probability that the model has one, two, or three segments at 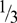 each, and 0 beyond that. We estimate *σ_e_* (the deviation of the expression Gaussian) to be the standard deviation of the known observations as described previously. We set both *σ_s_* (the stretching deviation) and *σ_a_* (the amplitude deviation) to be 10×(# genes)^−1^. Thus the three main components of the model have roughly similar influence.

We assemble queries by randomly subsampling time series in our data set. We assemble ten such queries from each treatment. We build each query by first selecting the number of observations in it, then choosing which time points will be represented, and finally picking an observation for each of these time points. The query sizes are chosen from a uniform distribution that ranges from one up to the number of observed times in the given treatment. The maximum size of a query is eight, although most consist of four or fewer observations. The time points are chosen uniformly as are the observations for each chosen time.

We then classify and align the query using all the other observations as the database. We preprocess both the query and the eleven database treatments using smoothing splines to reconstruct pseudo-observations at every four hours (starting at time zero, when all expression values are at the basal level). As before, we use the highest interpolation order possible in cases where there are too few observations for the prescribed one. We then align the query against all eleven treatments using our method. We return the database treatment with the highest scoring alignment, as defined by Equation 14. Because the alignment also maps each query time to a database treatment time, we can find the temporal error for any query time point. We thus calculate the average temporal error for the times in the original query in order to assess alignment error.

We consider several other alignment methods as baselines. We term the first baseline *one-segment generative*. This method is essentially the same as our multisegment generative alignment method, except that its alignments consist of only a single segment. It allows amplitude scaling and stretching, but only within its one segment pair.

The second control is traditional Euclidean dynamic time warping [3],[4]. Briefly, this method computes alignments by creating a matrix Г with elements defined recursively as
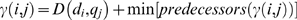
(20)where *D*(*d_i_*,*q_j_*) is the Euclidean distance between points *d_i_* and *q_j_* in the two series and *predecesssors*(*γ*,(*i*,*j*)) refers to the matrix elements adjacent to *γ*(*i*,*j*) with both indices less than or equal to *i* and *j* respectively. The first element *γ*(0,0) is just the Euclidean distance at time 0, and each other element *γ*(*i*,*j*) is the score of warping *d* from times 0 to *i* and *q* from 0 to *j*. We then create a normalized score matrix *Γ̅* where
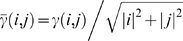
(21)


This makes it easy to compare warpings to different treatments, where one or the other dimension has been shorted.

Another control we consider is linear parametric warping. This is similar to the method explored by Bar-Joseph et al. [11], except that we make the assumption that the series are aligned at time zero. To find an alignment, we search possible slopes of the alignment line, and return the slope that results in the least average Euclidean distance between the query and the given database treatment.

Finally, we consider *correlation optimized warping* (COW) [14] as another baseline. This method takes as input two parameters: the number of warping segments *m* and a slack factor *s*. Both the query series and the database series are split into *m* segments. However while the segments of the query series are of equal length, the segment lengths of the database series may be up to *s* longer or shorter than an equal division would warrant. It is assumed that the starting and ending points of both series are aligned. The Pearson's correlation of each segment pair is calculated, and these are summed to score a given alignment. Dynamic programming is used to find the exact lengths of the database segments that maximize this value. We tried all values for *m* from one to ten together with all the values for *s* from zero to five. We report results for those (*m* = 10 and *s* = 5) that resulted in the highest accuracies.

**Figure 6:**
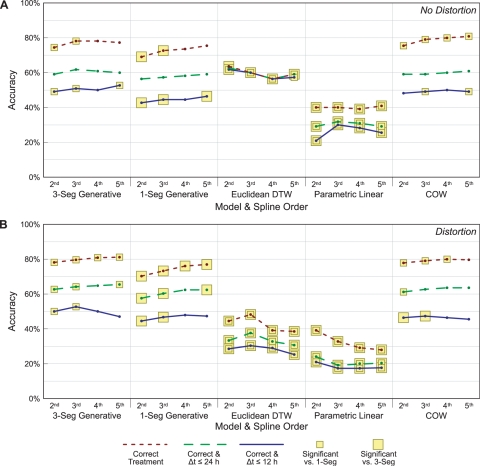
Classification and alignment accuracies for our generative method and others. The figure shows both when there is no temporal distortion (A), and when there is (B). The top lines represent treatment classification accuracy, while the bottom two lines add the criterion that the predicted times are within 24 and 12 hours respectively of the actual time, on average. Small highlights represent cases in which there is a significant difference in accuracy from the corresponding one-segment generative case (*p*≤0.05 with McNemar's *χ* ^2^ test), while the larger highlights show a significant difference from the three-segment model.

The results of these experiments are shown in Figure 6A. For each method the top line represents classification accuracy with different orders of splines, the middle line represents alignment accuracy by adding the criterion that the average time error in the mapping is less than or equal to 24 hours, and the bottom line shows alignment accuracy where this tolerance is decreased to 12 hours. Points highlighted with a small square are significantly different from the corresponding point using our one-segment generative model (*p*≤0.05) according to McNemar's *χ* ^2^ test. Likewise, the large square indicates a significant difference from the three-segment generative model.

The one-segment and three-segment models are only significantly different from each other in a handful of cases. Because we have added no distortion to the queries, the one-segment model should be sufficient to explain them. We might expect to see some degradation when using the three-segment model, as it is allowed much more freedom in where it places its segments. However, it seems that this is not the case; the three-segment model results in slightly higher accuracies. One explanation for this result is that the spline preprocessing does not create perfect reconstructions of the missing data, and the more expressive three-segment model is better at compensating for this error. Of the control methods, only COW is competitive with our generative method. There is no significant difference between its accuracy and that of our method. Euclidean dynamic time warping classifies fewer queries correctly than our method, although those it does tend to be aligned correctly. This is probably because it has a strong bias toward performing little warping.

To better test the utility of the multisegment model, we next consider distorting the query time series temporally. We use three different distortions. The first one doubles all times in the first 48 hours (i.e., it stretches the first part of the series), and then halves all times (plus an offset for the doubling) for the next 24 hours. The second distortion halves for the first 36 hours and then doubles for 60 hours. The third one triples for the first 60 hours and then thirds for another 20. It should be noted that not all the treatment observations extend this long in time. The short ones (e.g., those for which we only have measurements up to 24 or 48 hours) will thus not be distorted as much as the long ones.

Aside from the distortion, we perform the same experiment as before. We show the results in Figure 6B. In this experiment, the three-segment model results in more accurate classifications and alignments than the simpler one-segment model. Both DTW and the linear method appear brittle when confronted with distortions. Although our three-segment method significantly beats COW only when the strictest correctness criteria are used, the results shown are the best COW returned for a wide variety of parameters. We did not perform a similar parameter search for our own method.

One concern is that by adding distortion we could be changing the best classification of a given treatment. For example, maybe we would distort 10 µg/kg of TCDD in exactly the right way to make it look like 64 µg/kg. To address this concern, we have performed similar distortion experiments in which we align a distorted query series only to the database series that was used to generate it. The results of this experiment are qualitatively the same as those reported in Figure 6.

### Effect of Stretching and Amplitude Components

**Figure 7:**
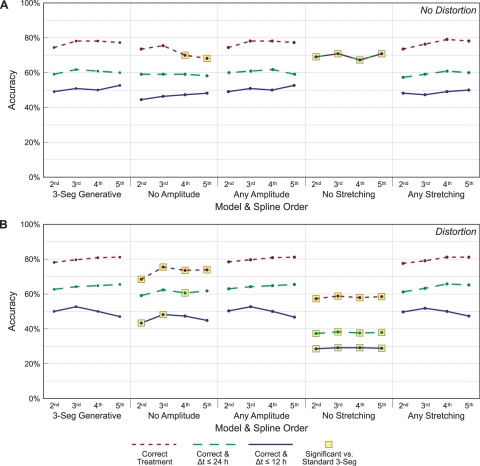
Classification and alignment accuracies when we have removed components of the model. The panels show distortion not present (A) and present (B). The first model is the three-segment generative model as before. The second disallows any amplitude changes at all, while the third allows any amplitude coefficient with no penalty to the score. Likewise, the fourth disallows stretching and the fifth allows any stretching without penalty. Highlights indicate a significant difference from the unaltered three-segment model (*p*≤0.05 with McNemar's *χ* ^2^ test).

We conduct further experiments to evaluate the importance of the stretching and amplitude components of our model. First, we conduct an experiment in which we effectively remove the amplitude component of our model by fixing the value of *a_i_* to 1.0 for all segments. With all of the probability mass on this single value, the log *P_a_*(*a_i_*) term in Equation 14 becomes zero. In a separate experiment, we set *σ_a_* = ∞, which makes all amplitude changes equally likely. Similarly, we perform experiments in which we force *s_i_* to 1.0 and set *σ_s_* = ∞. The results of these experiments are shown in Figure 7.

Totally disallowing either stretching or amplitude changes has an overall deleterious effect on the accuracy of the alignments. However there seems to be little negative effect in allowing stretching and amplitude changes but not penalizing for greater values. These results imply that the stretching and amplitude components of the model are valuable, but that the accuracy of the alignments is relatively insensitive to the actual penalties selected.

### Effect of Query Size and Number of Segments

We next consider a set of experiments in which we assess the accuracy of computed alignments as a function of the amount of data in the query. We restrict our experiments to a single treatment (41 observations of 1 µg/kg TCDD at eight time points), although other treatments yielded qualitatively similar results. We randomly pick out *n* observations from different times in the treatment to form each query. We use all the remaining observations in the treatment as the database. We interpolate both query and database series as before (every four hours), compute the best alignment using the one-segment and three-segment methods, and then assess alignment error. We do this 100 times for each value of *n*, which we vary from one to eight. We also vary the spline order from two to five, and repeat the experiment with the query times distorted (as in the last section) and not distorted. We perform paired, two-tailed *t*-tests on the alignment errors from the two methods in order to determine significant differences.

We expect the alignment error to generally decrease as we increase the query size. We also expect the one-segment method to perform slightly better when there is no distortion, and the three-segment method to be preferable when there is. However this latter behavior could be confounded for small query sizes, where the three-segment model may not have enough data to determine the segment parameters.

**Figure 8:**
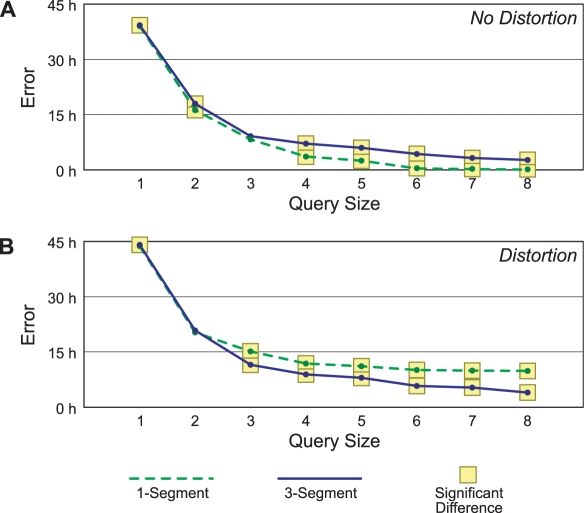
Average alignment error versus query size. The results shown in (A) have no temporal distortion, while those shown in (B) do. The dotted line represents the one-segment model, and the solid line represents the three-segment model, using third-order smoothing splines. Cases in which the two have significantly different results (*p*≤0.05 with a two-tailed Student's *t*-test) are highlighted.

The results when we interpolate with third-order splines are shown in Figure 8. (The other orders of spline yield substantially similar results.) For queries of size two or less, the one-segment model performs slightly better. Its average error is less than that of the three-segment model, by less than one hour. However as the query size grows larger, the expected results become more apparent. When there is no distortion, the one-segment model is adequate. When there is distortion, a multisegment model is clearly preferable.

**Figure 9:**
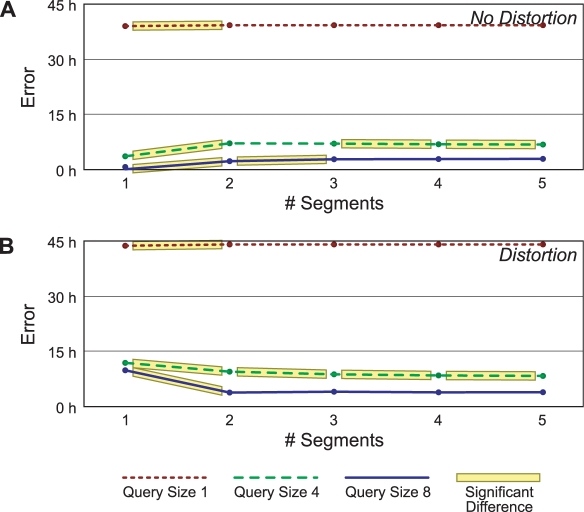
Average alignment error versus number of segments in the model. As before, the results in (A) have no temporal distortion while those in (B) do. From top to bottom, the lines of each panel show queries of size one, four, and eight, using third-order smoothing splines. Lines are highlighted in cases where adding a segment to the model makes a significant difference (*p*≤0.05 with a two-tailed Student's *t*-test).

We next consider the sensitivity of the accuracy of the multisegment method to the number of segments it is allowed to use in its alignments. We would like to know to what extent the alignment accuracy degrades as the method is allowed to use more segments than the optimal alignment requires. We conduct an experiment in which we vary the number of segments from one to five, with query sizes of only one, four, and eight. The results of this experiment are shown in Figure 9 for the third-order spline case. Here each line represents one of the query sizes, from one at the top to eight at the bottom. A highlighted part of a line shows a significant change in alignment accuracy when going from an *m*-segment model to an (*m*+1)-segment model.

Again, we see that in the data-rich situation, the best models are those that closely approximate the number of segments needed to simulate the temporal distortion (or lack thereof) applied to the query. In data-poor situations, the alignments of the one-segment method are as accurate as multisegment alignments. Significantly, the accuracy of the multisegment method is quite robust when it is allowed to use more segments than necessary. This is important, as in practice we will not generally know the correct number of segments in order to find the best alignment of a query and its best matching series in the database.

### An Alignment Example

**Figure 10:**
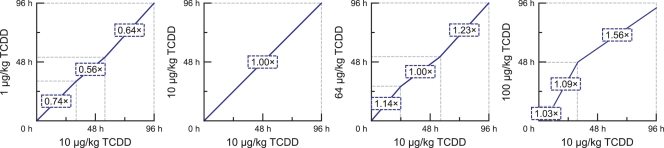
Alignments found by our multisegment method between four different dosages of TCDD. The boxed numbers on each segment represent the amplitude coefficient by which the expression levels of the 10 µg/kg segment are best multiplied in order to obtain the corresponding expression levels for the other treatment.

Finally, we consider calculating the alignments for four treatments that we know are closely related. Figure 10 illustrates the alignments computed by our method for a 10 µg/kg dose of TCDD to itself and three other dosages of the same chemical.

These alignments illustrate several interesting phenomena. First, they indicate that the overall amplitude of the response increases along with the dose. This effect is illustrated by the boxed numbers on the segments in Figure 10. Second, the 10 µg/kg and 64 µg/kg dosages induce similar responses, both in their amplitude and temporal evolution. Third, the alignment to the 100 µg/kg dosage suggests that the response induced by this treatment initially progresses more slowly than the responses caused by the lower doses. This somewhat surprising result and the abovementioned effects are consistent with the expression profiles for the highly expressed genes shown in Figure 4.

## Discussion

We have presented an approach for answering similarity queries among gene expression time series, and aligning those queries in time. Our approach employs spline models to interpolate sparse time series, and a novel method for time warping. We have investigated our approach in the context of a toxicogenomics application in which we would like to know which treatments in a database of well characterized chemicals are most similar to a given query treatment.

The work we have presented features several novel aspects and contributions.

- We have introduced a novel, *multisegment* alignment method for time series. This method offers more flexibility than linear alignment methods, yet is more constrained than the standard dynamic time warping approach. Our multisegment method is able to find accurate alignments in cases in which part, but not all, of the expression response occurs more slowly (rapidly) or has a smaller (greater) amplitude in one treatment than in a similar treatment.
- To account for the fact that we have sparse time series, we have investigated the use of a variant of B-splines we refer to as *smoothing* splines. Smoothing splines determine their control points from interpolations calculated with lower-order splines.
- We have empirically shown that our smoothing splines result in more accurate alignments than both conventional *intercepting* B-splines and a linear interpolation baseline.
- We have empirically demonstrated that our generative alignment method generally produces more accurate alignments and treatment classifications than other commonly used alignment methods, including conventional dynamic time warping, linear parametric, and correlation optimized warping.

There are several avenues of future work we plan to pursue. One is to address the time complexity of our multisegment algorithm, which is *O*(*n* ^5^), where *n* is the length of the series. Alignment to all eleven database series and subsequent classification currently take about a half hour to execute. By contrast, the time complexity of ordinary dynamic time warping is only *O*(*n* ^2^). When the calculations are restricted to the so-called *Sakoe-Chiba band*, a narrow band centered on the diagonal of the warping matrix, the time complexity approaches *O*(*n*) [20]. We would like to devise heuristics to speed up our multisegment method. For example, although shorting complicates the use of a Sakoe-Chiba band, it might be possible to restrict calculations in the warping space to some other shape, such as a cone. Alternatively, we could perform a first pass with the faster one-segment model, and then restrict the multisegment model to an area near it in warping space.

In addition, we have made two independence assumptions that we plan to revisit in future research. First, we have assumed that each gene is independent of all the others given the model. We expect that representing some gene dependencies would lead to more accurate classifications and alignments. Second, we assume that the measurements at each time point are independent of each other time point. We plan to investigate a Markov-model like approach that represents dependencies between neighboring time points.

## References

[1] Hayes K, Vollrath A, Zastrow G, McMillan B, Craven M (2005). EDGE: a centralized resource for the comparison, analysis and distribution of toxicogenomic information.. Mol Pharmacol.

[2] Ernst J, Nau G, Bar-Joseph Z (2005). Clustering short time series gene expression data.. Bioinformatics.

[3] Sakoe H, Chiba S (1978). Dynamic programming algorithm optimization for spoken word recognition.. IEEE Trans Acoust.

[4] Sankoff D, Kruskal J (1983). Time Warps, String Edits, and Macromolecules: The Theory and Practice of Sequence Comparison.

[5] Thomas R, Rank D, Penn S, Zastrow G, Hay K (2001). Identification of toxicologically predictive gene sets using cDNA microarrays.. Mol Pharmacol.

[6] Natsoulis G, Ghaoui LE, Lanckriet G, Tolley A, Leroy F (2005). Classification of a large microarray data set: algorithm comparison and analysis of drug signatures.. Genome Res.

[7] Lamb J, Crawford E, Peck D, Modell J, Blat I (2006). The connectivity map: using gene-expression signatures to connect small molecules, genes and disease.. Science.

[8] Aach J, Church G (2001). Aligning gene expression time series with time warping algorithms.. Bioinformatics.

[9] Criel J, Tsiporkova E (2006). Gene time expression warper: a tool for alignment, template matching and visualization of gene expression time series.. Bioinformatics.

[10] Liu X, Müller HG (2003). Modes and clustering for time-warped gene expression profile data.. Bioinformatics.

[11] Bar-Joseph Z, Gerber G, Gifford D, Jaakkola T, Simon I (2003). Continuous representations of time-series expression data.. J Comput Biol.

[12] Listgarten J, Neal R, Roweis S, Emili A, Saul L, Weiss Y, Bottou L (2005). Multiple alignment of continuous time series.. Advances in Neural Information Processing Systems 17.

[13] Gaffney SJ, Smyth P, Saul LK, Weiss Y, Bottou L (2005). Joint probabilistic curve clustering and alignment.. Advances in Neural Information Processing Systems 17.

[14] Nielsen NV, Carstensen JM, Smedsgaard J (1998). Aligning of single and multiple wavelength chromatographic profiles for chemometric data analysis using correlation optimised warping.. J Chromatogr A.

[15] Burge C, Karlin S (1997). Prediction of complete gene human genomic DNA.. J Mol Biol.

[16] Schmidler S, Liu J, Brutlag D (2000). Bayesian protein secondary structure.. J Comput Biol.

[17] Rogers D, Adams J (1989). Mathematical Elements Graphics.

[18] Altschul S (1991). Amino acid substitution matrices information theoretic perspective.. J Mol Biol.

[19] Durbin R, Eddy S, Krogh A, Mitchison G (1998). Sequence Analysis: Probabilistic Models of Proteins Acids.

[20] Ratanamahatana C, Keogh EJ (2005). Three myths time warping data mining.. Proceedings of SIAM Conference on Data Mining.

