# Correction: Similarity Queries for Temporal Toxicogenomic Expression Profiles

**DOI:** 10.1371/annotation/48ea9acf-4422-4792-b9a6-565de3e64f60

**Published:** 2008-11-25

**Authors:** Adam A. Smith, Aaron Vollrath, Christopher A. Bradfield, Mark Craven

This URL is missing a tilde. The correct URL reads: http://www.biostat.wisc.edu/~aasmith/catcode/

